# Reprogramming Roadblocks Are System Dependent

**DOI:** 10.1016/j.stemcr.2015.07.007

**Published:** 2015-08-13

**Authors:** Eleni Chantzoura, Stavroula Skylaki, Sergio Menendez, Shin-Il Kim, Anna Johnsson, Sten Linnarsson, Knut Woltjen, Ian Chambers, Keisuke Kaji

**Affiliations:** 1 MRC Centre for Regenerative Medicine, University of Edinburgh, Edinburgh BioQuarter, 5 Little France Drive, Edinburgh EH16 4UU, Scotland; 2 Department of Biosystems Science and Engineering, ETH Zurich, 4058 Basel, Switzerland; 3 Center for iPS Cell Research and Application (CiRA), Kyoto University, Kyoto 606-8507, Japan; 4 Laboratory for Molecular Neurobiology, Department of Medical Biochemistry and Biophysics, Karolinska Institute, Scheeles väg 1, 171 77 Stockholm, Sweden; 5 Hakubi Center for Advanced Research, Kyoto University, Kyoto 606-8501, Japan

## Abstract

Since the first generation of induced pluripotent stem cells (iPSCs), several reprogramming systems have been used to study its molecular mechanisms. However, the system of choice largely affects the reprogramming efficiency, influencing our view on the mechanisms. Here, we demonstrate that reprogramming triggered by less efficient polycistronic reprogramming cassettes not only highlights mesenchymal-to-epithelial transition (MET) as a roadblock but also faces more severe difficulties to attain a pluripotent state even post-MET. In contrast, more efficient cassettes can reprogram both wild-type and *Nanog*^−**/**−^ fibroblasts with comparable efficiencies, routes, and kinetics, unlike the less efficient reprogramming systems. Moreover, we attribute a previously reported variation in the N terminus of KLF4 as a dominant factor underlying these critical differences. Our data establish that some reprogramming roadblocks are system dependent, highlighting the need to pursue mechanistic studies with close attention to the systems to better understand reprogramming.

## Highlights

• Distinct reprogramming cassettes yield different reprogramming intermediates
• MET is not a major rate-limiting step in reprogramming with high KLF4 expression
• A lack of endogenous *Nanog* becomes trivial in efficient reprogramming systems
• Roadblocks toward iPSCs depend on the reprogramming systems

## Graphical Abstract

**Figure:**
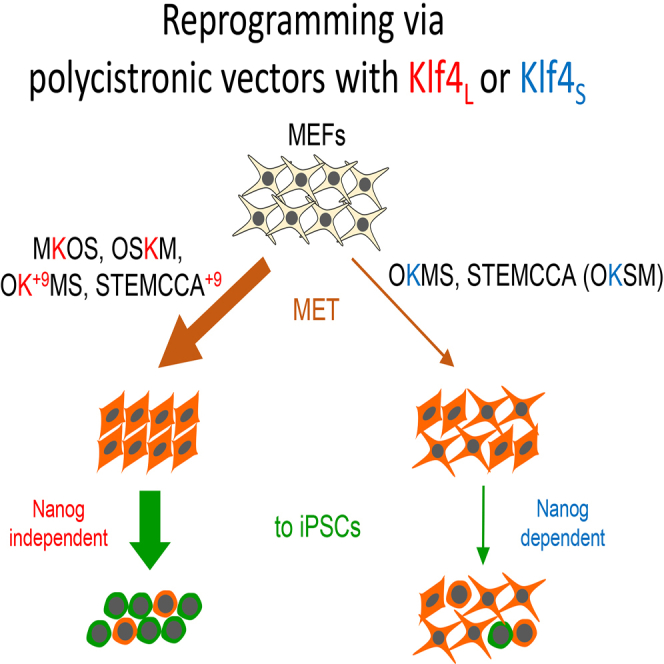

## Teaser

In this article, Kaji and colleagues demonstrate that different reprogramming systems with distinct reprogramming efficiencies yield clearly distinguishable intermediate populations and, thus, potentially biased mechanistic views. For example, mesenchymal-to-epithelial transition and lack of endogenous *Nanog* are obstacles for iPSC generation only in inefficient reprogramming systems. This work highlights the importance of re-assessing molecular mechanisms of reprogramming in the literature heeding the reprogramming systems.

## Introduction

Ever since the first generation of induced pluripotent stem cells (iPSCs) using retroviral vectors for *Oct4*, *Sox2*, *Klf4*, and *c-Myc* (O, S, K, and M, respectively) (Takahashi and Yamanaka, 2006), many researchers have focused on understanding the reprogramming mechanism for a more efficient and rapid generation of iPSCs. To this end, it is essential to understand the molecular roadmaps toward successful reprogramming, to identify bottlenecks, and to develop strategies to overcome these obstacles. Recently, a few detailed reprogramming roadmaps have been described from time course gene expression analyses as well as cell-surface-marker-based reprogramming intermediate subpopulation analyses (Hussein et al., 2014; O’Malley et al., 2013; Polo et al., 2012). In this context, we have reported that monitoring expression changes of CD44, ICAM1, and a *Nanog*-GFP reporter enables the tracking of the successful progression of reprogramming using a 2A-peptide-linked MKOS polycistronic reprogramming cassette (M-K-O-S in this order) (O’Malley et al., 2013). Reprogramming intermediates isolated based on their CD44/ICAM1/*Nanog*-GFP profile demonstrated an increasing probability to reach a pluripotent state concordant with the stage of progression defined by the markers. However, whether the roadmaps change when different reprogramming systems that have distinct reprogramming efficiencies are used has not been addressed yet.

Reduction of heterogeneity during reprogramming is critical for revealing molecular roadmaps and precise mechanistic analysis. In this regard, the use of 2A peptides constituted a significant improvement (Carey et al., 2009; Kaji et al., 2009; Okita et al., 2008; Sommer et al., 2009; Yusa et al., 2009). Polycistronic cassettes carrying all four factors linked with 2A peptides have simplified the reprogramming procedure and established a consistent reprogramming factor stoichiometry. On the other hand, it has also been shown that the use of distinct polycistronic vectors could result in different reprogramming efficiencies (Okita et al., 2008). This suggests that reprogramming with different polycistronic cassettes, which may have distinct stoichiometry and/or expression levels of the Yamanaka factors, could face various degrees and/or kinds of roadblocks during reprogramming.

Reprogramming in the absence of *Nanog* is one example where cells face a significant roadblock and/or deviate from the original route. *Nanog* belongs to the core transcription factors of the pluripotency transcription network and has been shown to be important for maintenance and induction of pluripotency (Chambers et al., 2003; Mitsui et al., 2003; Silva et al., 2009). Recent studies have shown that *Nanog* null mouse embryonic fibroblasts (MEFs) can give rise to iPSCs only in the presence of Vitamin C (VitC) (Schwarz et al., 2014) or with 100-fold less efficiency compared to wild-type (WT) MEFs in the absence of VitC (Carter et al., 2014). Nevertheless, *Nanog* is still largely considered important for iPSC generation, and it has not been addressed yet whether the *Nanog*^−/−^ cells that fail to become iPSCs halt at a certain point of the reprogramming route or divert to a different cell state.

Taking advantage of a cell surface marker profiling system and efficiently reprogrammable MEFs, we investigated reprogramming routes and roadblocks using several polycistronic cassettes and *Nanog*^−/−^ MEFs. We demonstrate that the pattern of cell surface marker changes is different when reprogramming cassettes with distinct reprogramming efficiencies are used. The majority of reprogramming intermediates derived using a less efficient reprogramming cassette is trapped in partially reprogrammed states, concealing the route that the few iPSCs followed. On the contrary, efficient reprogramming cassettes can reprogram even *Nanog*^−/−^ MEFs with efficiency, routes, and kinetics comparable to those of WT MEFs. Therefore, we highlight the necessity to take into consideration the reprogramming system when mechanistic analyses are performed.

## Results

### Polycistronic Cassettes with Distinct Reprogramming Phenotypes

**Figure 1:**
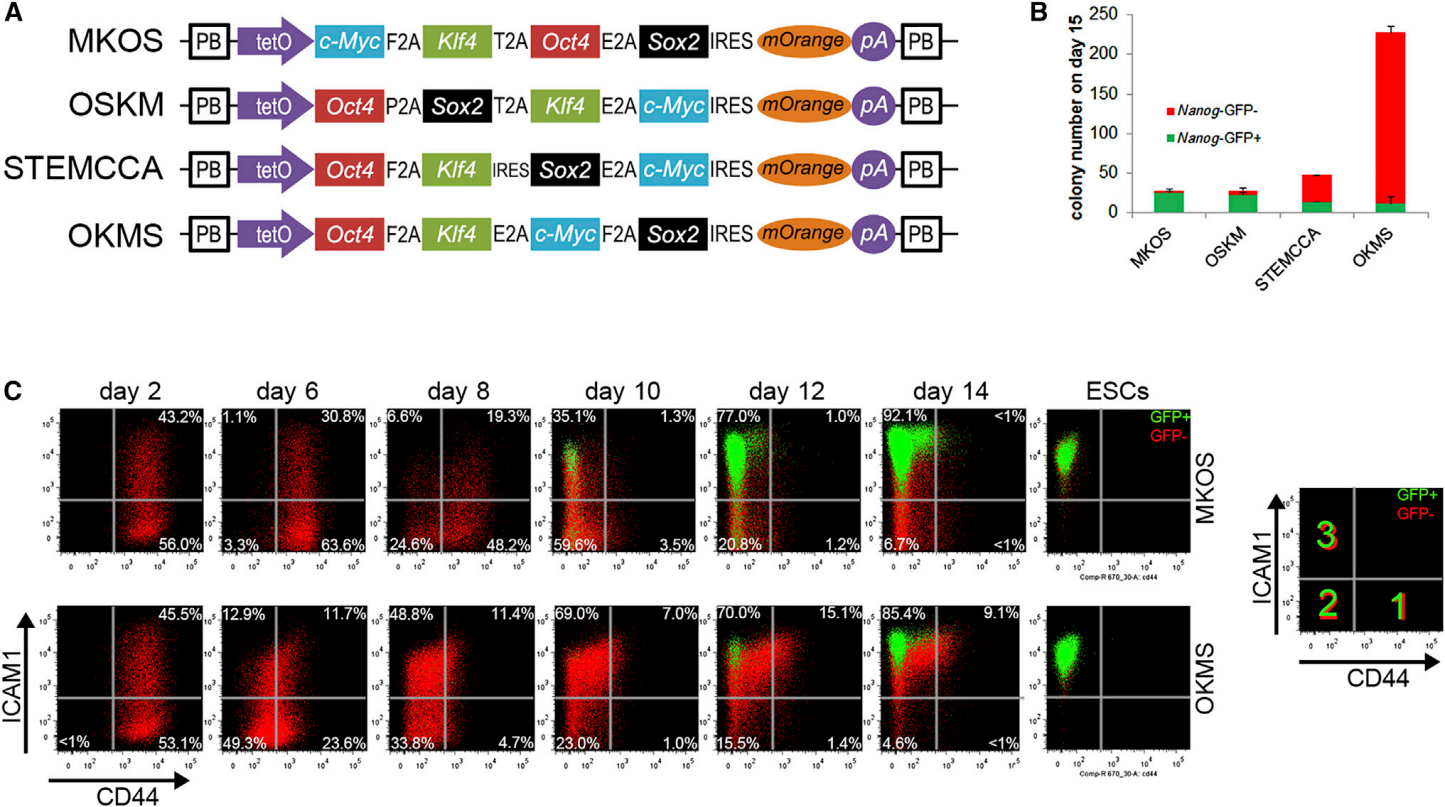
PB Reprogramming with Different Polycistronic Cassettes (A) Structures of PB transposons carrying different polycistronic cassettes used in this study. (B) Numbers of *Nanog*-GFP^+/−^ colonies 15 days after reprogramming using the PB MKOS, OSKM, STEMCCA, or OKMS transposons. Error bars represent SD; n = 3 independent experiments. (C) CD44, ICAM1, and *Nanog*-GFP expression changes in PB reprogramming with the MKOS or OKMS cassette. Red indicates *Nanog*-GFP^−^, and green indicates *Nanog*-GFP^+^. Gates 1, 2, and 3 indicate ICAM1^low^/CD44^high^, ICAM1^low^/CD44^low^, and ICAM1^high^/CD44^low^, respectively, as indicated in the right panel.

Several polycistronic cassettes carrying the four Yamanaka factors have been used for the generation of iPSCs. We previously performed a detailed analysis of MKOS-induced reprogramming routes using CD44 and ICAM1 cell surface markers in combination with a gene-targeted *Nanog*-GFP reporter (O’Malley et al., 2013). Carey et al. (2011) demonstrated that iPSC lines derived with the OSKM cassette tend to have better chimera contribution when injected into blastocysts than those derived with the STEMCCA (OKSM) cassette in the absence of VitC. Thus, we performed piggyBac (PB)-transposon-mediated reprogramming with the aforementioned MKOS, OSKM, and STEMCCA cassettes to compare their potentials to give rise to iPSC colonies (Figure 1A). We also included the OKMS cassette in our analysis (Kim et al., 2015), generated from the OKS cassette that showed the highest reprogramming efficiency among other polycistronic cassettes containing three reprogramming factors, *Oct4*, *Sox2*, and *Klf4* (Okita et al., 2008). Indeed, the OKMS cassette gave rise to a much larger number of colonies with an embryonic stem cell (ESC)-like morphology when compared to the other constructs (Figure 1B). However, these colonies displayed limited activation of the *Nanog*-GFP reporter, suggesting that cells in most of the colonies were facing significant obstacles in reaching a fully pluripotent state (Figure 1B). The high proportion of *Nanog*-GFP^−^ partially reprogrammed colonies was also observed when the STEMCCA cassette was used, albeit to a lesser extent (Figure 1B). Most of the colonies in both MKOS and OSKM reprogramming, which previously demonstrated similar CD44 and ICAM1 expression changes (O’Malley et al., 2013), had become *Nanog*-GFP^+^ by day 15 (Figure 1B). We decided to focus on the comparison between MKOS and OKMS cassettes, given that they displayed the most striking differences in *Nanog*-GFP activation. Further differences stood out when we analyzed CD44, ICAM1, and *Nanog*-GFP expression changes during PB-mediated reprogramming (Figure 1C). As previously shown (O’Malley et al., 2013), reprogramming with MKOS demonstrated clear stepwise changes in CD44 and ICAM1 expression. In particular, we observed loss of the ICAM1^high^ population (gate 1 in Figure 1C, days 6–10) and downregulation of CD44 (gate 2 in Figure 1C, days 8–10), followed by the regain of ICAM1 expression (gate 3 in Figure 1C, days 8–14) accompanied by *Nanog*-GFP induction. In contrast, OKMS reprogramming displayed a very distinct flow cytometry profile. The majority of cells had already downregulated CD44 by day 6, without any clear change in ICAM1 expression, while some cells remained CD44/ICAM1 double-positive even on day 14 (Figure 1C). Finally, *Nanog*-GFP^+^ cells appeared only in the CD44^low^ICAM1^high^ gate (gate 3 in Figure 1C) where ESCs and fully reprogrammed iPSCs are found, in contrast to MKOS reprogramming in which some cells had gained *Nanog*-GFP expression before reaching gate 3 (day 10 and day 12 in Figure 1C) (O’Malley et al., 2013). These data suggest that reprogramming systems that allow expansion of partially reprogrammed cells demonstrate distinct CD44/ICAM1 expression changes.

### MKOS/OKMS Transgenic MEF Reprogramming System

**Figure 2:**
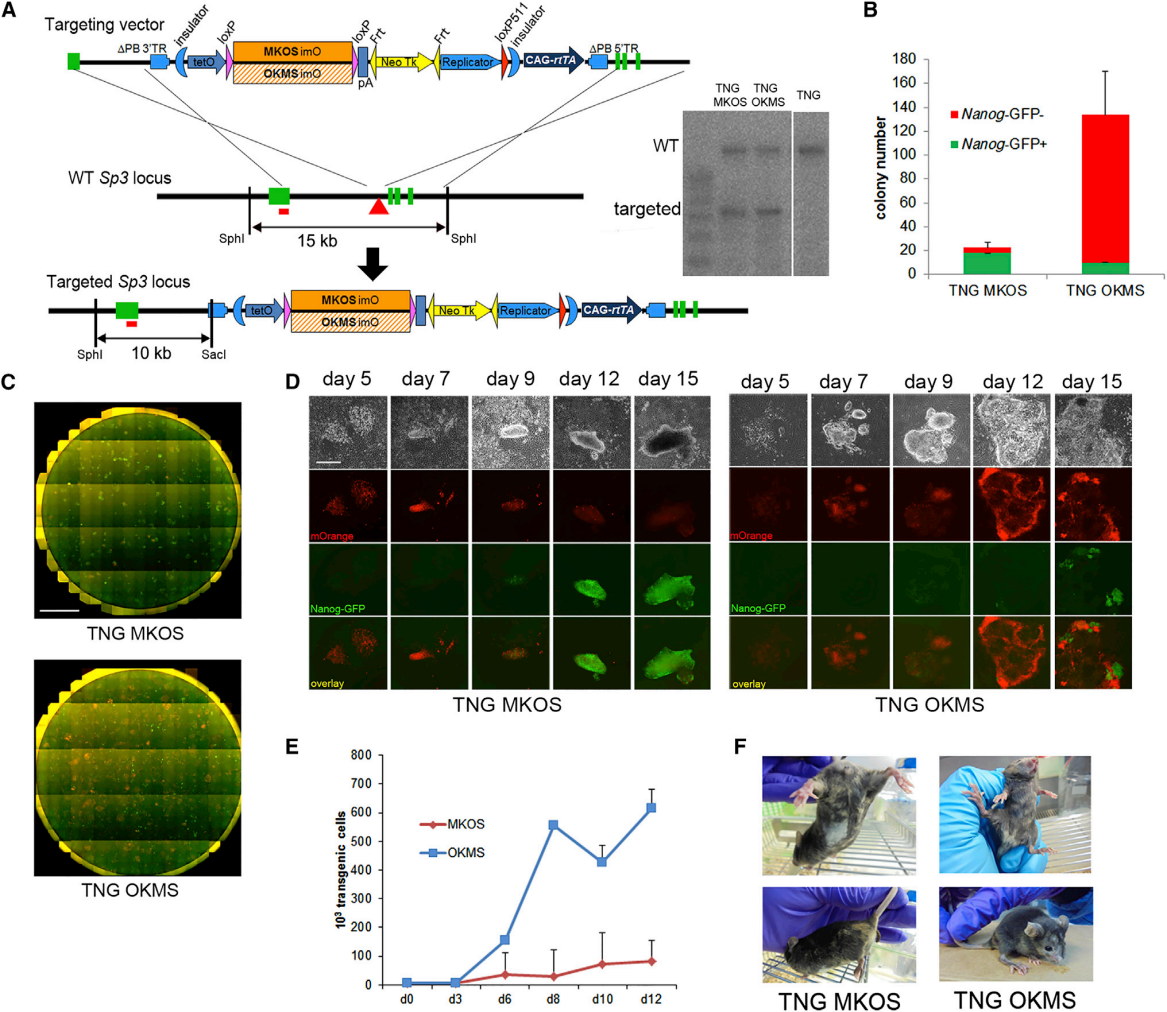
TNG MKOS/OKMS MEF Reprogramming System (A) The *Sp3* locus targeting scheme and Southern blot analysis. The green boxes represent *Sp3* exons 1–4 from the right side. The red triangle in the third intron indicates the PB transposon integration site identified in the D6s4B5 iPSC line. SacI/SphI double-genome digestion yielded WT 15 kb and targeted 10-kb fragments detected by the probe indicated as a red bar. pA, poly(A) signal. (B) Number of *Nanog*-GFP^+^/GFP^−^ colonies on day 15 of TNG MKOS/OKMS reprogramming. Error bars represent SD; n = 3 independent experiments. (C) Whole-well merged images of mOrange (red) and *Nanog*-GFP (green) on day 15 of TNG MKOS/OKMS reprogramming. Scale bar, 7 mm. (D) Tracking images of a typical TNG MKOS or OKMS reprogramming colony from days 5 to 15. Scale bar, 500 μm. (E) Tg cell numbers during TNG MKOS/OKMS reprogramming. Error bars represent SD; n = 3 independent experiments. (F) Chimeric mice generated with TNG MKOS or OKMS iPSC lines.

For a more precise comparison between MKOS and OKMS reprogramming, we generated a transgenic (Tg) MEF reprogramming system using all-in-one gene targeting vectors with doxycycline-inducible reprogramming factors (Figure 2A). In the previously reported reprogrammable MEF/mouse systems, either the *Col1a1* or the *Rosa26* locus was used to insert the OSKM or STEMCCA reprogramming cassette (Carey et al., 2010; Haenebalcke et al., 2013; Stadtfeld et al., 2010). The *Col1a1* systems require reverse tetracycline-controlled transactivator (rtTA) expression from another locus such as the *Rosa26* locus; therefore, two rounds of gene targeting are necessary (Carey et al., 2010; Stadtfeld et al., 2010). When MEFs from the *Rosa26* system carry both the reprogramming and an rtTA cassette at the same locus, transgene induction occurred only in 9% (heterozygous) or 15% (homozygous) of MEFs, indicating that the *Rosa26* locus is not optimal for placement of the doxycycline-inducible transgenes (Haenebalcke et al., 2013). In our system, a vector carrying a doxycycline-inducible MKOS or OKMS reprogramming cassette along with a CAG-promoter (chicken β-actin promoter with cytomegalovirus [CMV] enhancer)-driven rtTA cassette was targeted into the third intron of the *Sp3* gene of the TNG ESC line, which contains a *Nanog*-GFP reporter (Chambers et al., 2007), resulting in TNG MKOS or TNG OKMS ESCs. The *Sp3* locus was identified in an iPSC line with a single integration of the PB MKOS reprogramming transposon, D6s4B5, previously used for efficient secondary reprogramming (O’Malley et al., 2013). TNG MKOS or TNG OKMS ESCs were used to generate chimeric embryos, from which Tg MEFs (TNG MKOS or TNG OKMS MEFs) were prepared. Tg MEFs could be identified by culturing cells in the presence of doxycycline, resulting in the expression of an mOrange reporter linked to the reprogramming cassettes with an *ires* sequence (imO; Figure 2A). Similarly to the PB MKOS reprogramming, almost all the colonies from TNG MKOS MEFs had gained robust *Nanog*-GFP expression by day 12 (Figures 2B–2D). In contrast, TNG OKMS MEFs showed increased proliferation of Tg cells and gave rise to more ESC-like colonies. However, these colonies bore heterogeneous activation of the *Nanog*-GFP reporter (Figures 2B–2E), consistent with the previously observed PB reprogramming phenotype using OKMS cassettes (Figure 1B). Importantly, *Nanog*-GFP^+^ cell lines established from both TNG MKOS and TNG OKMS reprogramming could contribute to live chimeras (Figure 2F), demonstrating that both MKOS and OKMS systems could generate bona fide iPSCs.

### OKMS Reprogramming-Specific Roadblocks

**Figure 3:**
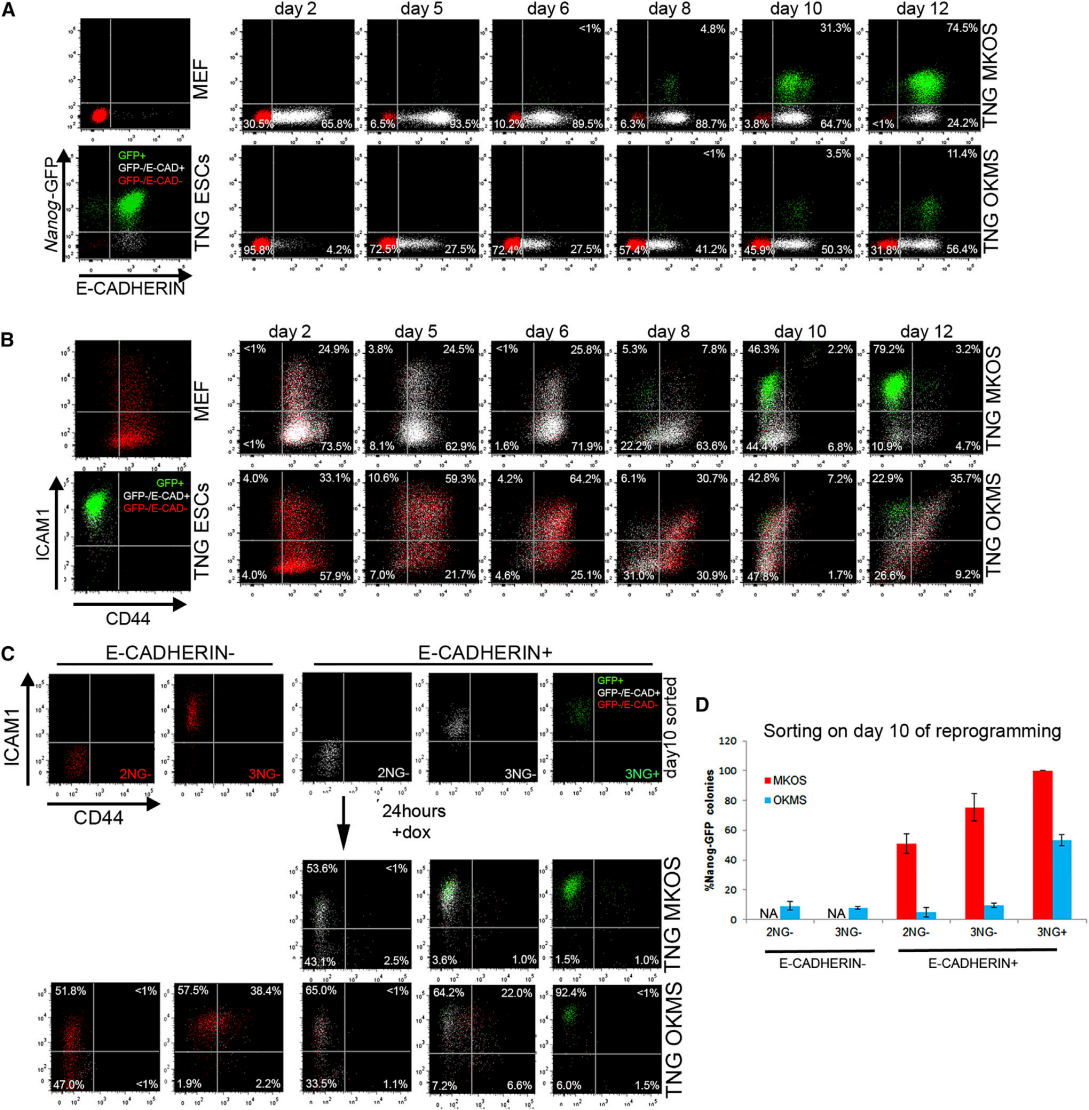
Inefficient Reprogramming Progression of OKMS Reprogramming Intermediates (A) E-CAD and *Nanog*-GFP expression changes during TNG MKOS/OKMS reprogramming. Red indicates E-CAD^−^*Nanog*-GFP^−^, white indicates E-CAD^+^*Nanog*-GFP^−^, and green indicates E-CAD^+^*Nanog*-GFP^+^. (B) CD44 and ICAM1 expression changes during TNG MKOS/OKMS reprogramming with E-CAD, *Nanog*-GFP expression color codes in (A). (C) Flow cytometry analysis of sorted day-10 E-CAD^−/+^ 2NG− (*Nanog*-GFP^−^ CD44^−^ ICAM1^−^), 3NG^−^ (*Nanog*-GFP^−^ CD44^−^ ICAM1^+^), and 3NG^+^ (*Nanog*-GFP^+^ CD44^−^ ICAM1^+^) cells after a 24-hr culture. dox, doxycycline. (D) E-CAD^−/+^ 2NG^−^, 3NG^−^, and 3NG^+^ (*Nanog*-GFP^+^ CD44^−^ ICAM1^+^) cells on day 10 were seeded at clonal density, and *Nanog*-GFP^+^ iPSC colonies were counted after 10 days of further culture. The graph depicts the relative *Nanog*-GFP^+^ CFA compared to that of MKOS 3NG^+^ cells. Error bars represent SD; n = 3 independent experiments.

In order to characterize the intermediate subpopulations during MKOS and OKMS reprogramming, we analyzed the expression changes of E-cadherin (E-CAD; also known as CDH1), CD44, ICAM1, and *Nanog*-GFP with flow cytometry (Figures 3A and 3B). E-CAD is a hallmark of mesenchymal-to-epithelial transition (MET) that is an essential step in the early stage of reprogramming from MEFs to iPSCs. Factors that facilitate MET have been reported to enhance reprogramming efficiency (Samavarchi-Tehrani et al., 2010; Li et al., 2010). As we previously reported (O’Malley et al., 2013), ∼90% of cells undergoing MKOS reprogramming were E-CAD^+^ by day 5, indicating that MET is not a major barrier in MKOS reprogramming (Figures 3A and 3B, upper panels). In contrast, during OKMS reprogramming, only about 45% of *Nanog*-GFP^−^/mOrange^+^ cells expressed E-CAD even on day 10, highlighting that the difficulty to go through MET during reprogramming is system dependent (Figures 3A and 3B, bottom panels). Next, we aimed to compare how the pre- and post-MET populations progress toward an iPSC state in MKOS and OKMS reprogramming. For this purpose, we sorted E-CAD^+/−^*Nanog*-GFP^−^ reprogramming intermediates from the CD44^low^ICAM1^low^ (2NG^−^) and CD44^low^ICAM1^high^ (3NG^−^) gates on day 10 (Figure S1A) and plated them in reprogramming conditions. E-CAD^−^ populations, which were abundant only in OKMS reprogramming, had gained neither E-CAD nor *Nanog*-GFP expression 24 hr after sorting (Figure 3C). Both MKOS and OKMS E-CAD^+^ 2NG^−^ cells progressed to the 3NG^−^ gate in a similar manner. However, OKMS 3NG^−^ cells produced only 2.5% *Nanog*-GFP^+^ cells 24 hr after sorting, whereas their MKOS counterparts yielded 21.9% *Nanog*-GFP^+^ cells (Figure 3C). Even 72 hr after sorting, only 14% *Nanog*-GFP^+^ cells were observed from the OKMS E-CAD^+^ 3NG^−^ population (Figure S1B). Accordingly, pre- or post-MET 2NG^−^ and 3NG^−^ cells sorted from OKMS reprogramming hardly formed any iPSC colonies when seeded at clonal density and cultured for a further 10 days in reprogramming conditions (Figure 3D). On the other hand, their MKOS counterparts demonstrated increasing colony formation ability (CFA) from the 2NG^−^ to the 3NG^−^ stage (Figure 3D) (O’Malley et al., 2013). Similarly, OKMS intermediates sorted on day 8 of reprogramming had reduced CFA, indicating that the majority of them had lower potential to form iPSCs, regardless of the timing of the analysis (Figure S1C). However, even in OKMS reprogramming, *Nanog*-GFP^+^ cells from the CD44^low^ICAM1^high^ gate (3NG^+^ cells) had increased CFA, reaching about 60% of the MKOS 3NG^+^ cells (Figure 3D). These data indicate that intermediate populations from MKOS and OKMS reprogramming, even with the same E-CAD, CD44, and ICAM1 expression patterns, had very distinct probabilities to progress toward iPSCs before gaining *Nanog*-GFP expression. It is likely that the majority of OKMS intermediates face more severe challenges than MKOS intermediates, and only very few cells can reach a pluripotent state.

### RNA-Sequencing Analysis of MKOS/OKMS Reprogramming Intermediates

**Figure 4:**
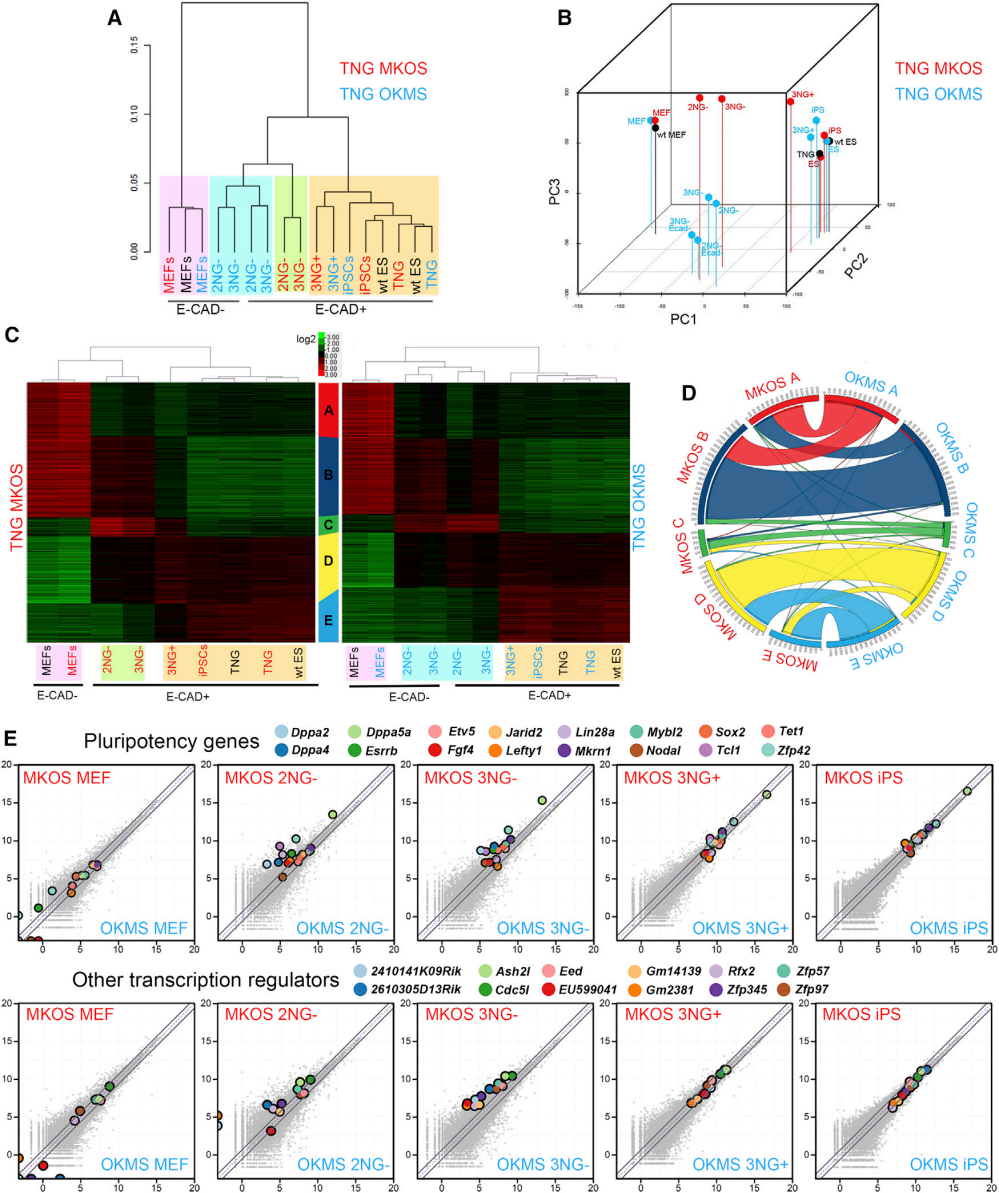
Distinct Gene Expression Profiles of MKOS/OKMS Reprogramming Intermediates (A) Hierarchical clustering of replicate averages with all genes. (B) PCA. Red or blue dots represent cells with MKOS or OKMS cassettes, respectively. Black dots represent cells without the reprogramming cassettes. (C) Expression heatmaps of MKOS/OKMS reprogramming with hierarchical clustering using DEGs, which were grouped to five clusters with distinct expression dynamics. (D) A chord diagram demonstrating three cross-classified DEG groups between MKOS A and OKMS B (MA_OB), MKOS B and OKMS A (MB_OA), and MKOS D and OKMS E (MD_OE). (E) Whole-transcriptome scatterplots highlighting the pluripotency genes (upper panels) and other transcription regulators (lower panels) identified in the MD_OE DEGs. The gray diagonal lines represent 1.5-fold differences in the expression levels.

To further characterize the 2NG^−^ and 3NG^−^ intermediate populations in MKOS/OKMS reprogramming, we performed RNA-sequencing analysis including WT/MKOS/OKMS MEFs, 3NG^+^ cells, and doxycycline-independent iPSCs from MKOS/OKMS reprogramming, as well as WT, TNG, and TNG MKOS/OKMS ESCs. Hierarchical clustering (average of replicates, all genes) revealed four major branches: (1) MEFs; (2) TNG OKMS E-CAD^+/−^, 2NG^−^, and 3NG^−^; (3) TNG MKOS E-CAD^+^, 2NG^−^, and 3NG^−^; and (4) TNG MKOS/OKMS 3NG^+^, iPSCs, and ESCs (Figure 4A). Principal-component analysis (PCA) with all genes clearly showed that MKOS reprogramming intermediates (2NG^−^ and 3NG^−^) were distinct from E-CAD^+^ and from E-CAD^−^ 2NG^−^ and 3NG^−^ cells in OKMS reprogramming (Figure 4B), in agreement with their different CFAs (Figure 3D). In contrast to the *Nanog*-GFP^−^ intermediates and consistent with the increased colony formation efficiency, OKMS *Nanog*-GFP^+^ (3NG^+^) cells clustered together with MKOS 3NG^+^ as well as iPSCs and ESCs (Figure 4B). Next, we identified differentially expressed genes (DEGs; false discovery rate ≤ 0.05) by comparing neighboring samples of the MKOS and OKMS reprogramming samples, respectively, and assigned each DEG to one of five groups (A–E) with distinct expression dynamics as defined by k-means clustering (Figure 4C). Most of the DEGs in MKOS and OKMS reprogramming overlap (6,126 of a total 7,291 DEGs in MKOS and 7,418 DEGs in OKMS), and their distribution into the five categories was also similar (Figure 4D). However, there were three large groups of genes that displayed different dynamics between MKOS and OKMS: (1) DEGs that displayed delayed downregulation in the OKMS reprogramming, i.e., 28.7% (435) of MKOS class A DEGs (1,514) overlapped with OKMS class B DEGs (MA_OB DEGs); (2) DEGs that had delayed downregulation in the MKOS reprogramming, i.e., 25.4% (579) of MKOS class B DEGs (2278) overlapped with OKMS class A DEGs (MB_OA DEGs); and, finally, (3) DEGs that displayed delayed upregulation in the OKMS reprogramming, i.e., 32.6% (612) of MKOS class D DEGs (1,873) overlapped with OKMS class E DEGs (MD_OE DEGs). Names of all DEGs in MKOS or OKMS reprogramming and the cross-overlapping DEGs are shown in Table S1. The most enriched gene ontology (GO) terms in the cross-classified MA_OB DEGs were “regulation of transcription” and “pattern specification process,” with Benjamini FDRs of 7.5 × 10^−3^ and 6.5 × 10^−3^, respectively (Benjamini and Hochberg, 1995). Genes with these GO terms include *Hoxa2*, *Hoxb5*, *Hoxc10*, and *Hoxd10*, and these genes associated with body pattern formation were downregulated more rapidly in MKOS reprogramming (Figure S2; Table S1). GO terms with the highest enrichment in MA_OB DEGs were “actin binding” and “cytoskeletal protein binding” (both with Benjamini 1.4 × 10^−11^). Genes with these highly enriched GO terms includes *Aif1l*, *Cap1*, *Capg*, *Capzb*, *Cnn1*, *Myo1c*, and *Myo1d* and were, indeed, more slowly downregulated in MKOS reprogramming (Figure S2; Table S1). It was surprising that downregulation of some genes occurred more slowly in the more efficient MKOS reprogramming system. Roles of these genes in reprogramming might be worth investigating in the future. MD_OE DEGs were rich in genes with GO terms associated with cell cycles and transcription, including multiple pluripotency genes (Figure S2; Table S1). Gene expression scatterplots revealed that many of these transcription factors were differentially expressed (>1.5-fold) in the 2NG^−^ and 3NG^−^ intermediate subpopulations, suggesting a potential contribution to the distinct probability to form iPSC colonies (Figure 4E). Interestingly, both the PCA and the heatmap demonstrated that MKOS 3NG^+^ cells still maintained some characters of 3NG^−^ cells, but OKMS 3NG^+^ cells were very distinct from 3NG^−^ cells and almost indistinguishable from iPSCs/ESCs (Figures 4B and 4C). These data indicated that the majority of OKMS 3NG^−^ cells were trapped in a partially reprogrammed state, and the transition to a fully reprogrammed state was difficult to track in the OKMS reprogramming system. It is not clear whether the few successfully reprogrammed OKMS 3NG^+^ cells came sporadically from the trapped state or whether a very small number of cells in OKMS reprogramming experienced gene expression changes as in MKOS reprogramming. Our analyses highlight the importance of characterizing the efficiency of cellular state transitions in conjunction with gene expression profiling when investigating reprogramming routes.

### The N-Terminal Nine Amino Acids of *Klf4* in the Polycistronic Reprogramming Cassettes Determine the Marker Expression Changes

**Figure 5:**
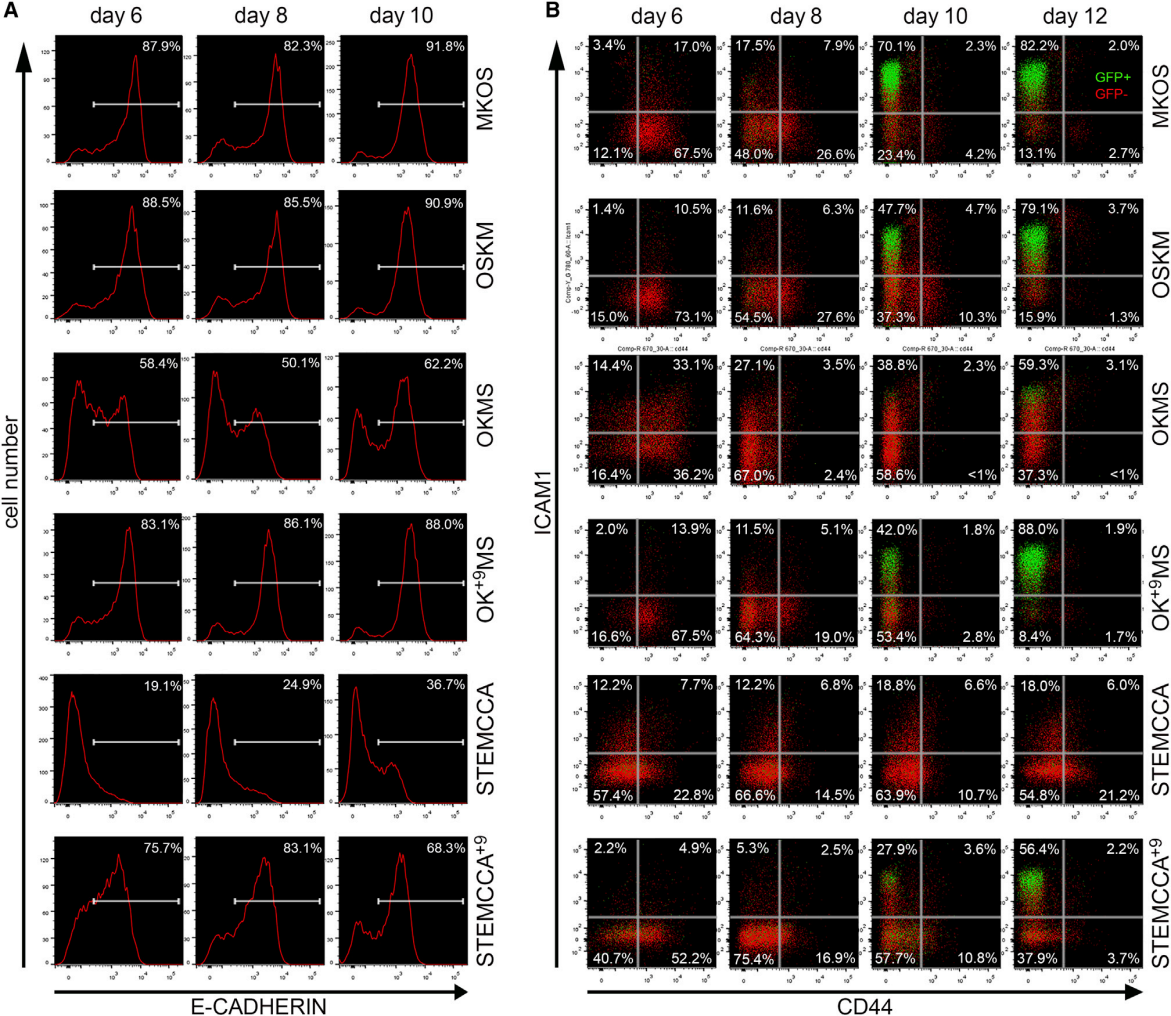
*Klf4*_L_ Restores Surface Marker and *Nanog*-GFP Expression Patterns during Reprogramming (A) E-CAD upregulation in PB reprogramming with the MKOS, OSKM, OKMS, OK^+9^MS, STEMCCA, and STEMCCA^+9^ cassettes. (B) CD44, ICAM1, and *Nanog*-GFP expression in MKOS, OSKM, OKMS, OK^+9^MS, STEMCCA, and STEMCCA^+9^ reprogramming. Red indicates *Nanog*-GFP^−^, and green indicates *Nanog*-GFP^+^.

While this manuscript was under revision, Kim et al. (2015) reported that the currently available polycistronic reprogramming cassettes containing four Yamanaka factors can be classified into two groups: those carrying a long *Klf4* (*Klf4*_*L*_, currently annotated as full length) or those carrying a short *Klf4* (*Klf4*_*S*_, missing the nine N-terminal amino acids of *Klf4*_*L*_) (Figure S3A). Interestingly, *Klf4*_*S*_ produced less protein compared to *Klf4*_*L*_ when used in the polycistronic cassettes (Figure S3B) (Kim et al., 2015). The polycistronic reprogramming cassettes containing *Klf4*_*S*_, such as OKMS, had lower reprogramming efficiency, which could be rescued by replacing *Klf4*_*S*_ with *Klf4*_*L*_ (OK^+9^MS) (Kim et al., 2015). Thus, we investigated whether the aforementioned differences in the expression patterns of E-CAD, CD44, and ICAM1 were also due to the difference in *Klf4*. Strikingly, reprogramming with OK^+9^MS and STEMCCA^+9^, carrying the *Klf4*_*L*_, demonstrated expression changes of all those markers, as well as *Nanog*-GFP, similar to those of MKOS reprogramming (Figures 5A and 5B). Recently, Nishimura et al. (2014) demonstrated that reducing the amount of KLF4 using a drug-inducible protein stabilization system resulted in the generation of partially reprogrammed cells trapped at different stages of reprogramming. In addition, *Klf4* is involved in promoting MET (Li et al., 2010). Our data demonstrate that clear stepwise CD44 and ICAM1 expression changes during reprogramming are also associated with the robust expression of *Klf4*.

### Lack of *Nanog* Does Not Constitute a Roadblock in MKOS Reprogramming

CD44 and ICAM1 markers revealed distinct molecular signatures between MKOS and OKMS reprogramming intermediates. Thus, we decided to further apply this marker analysis where significant roadblocks or route deviations were expected; namely, *Nanog*^−/−^ MEF reprogramming. *Nanog* has been reported to be essential for the generation of iPSCs (Silva et al., 2009), and overexpression of *Nanog* along with the Yamanaka factors increased efficiency and accelerated kinetics of reprogramming (Hanna et al., 2009). Two groups have recently reported the successful generation of *Nanog*^−/−^ iPSCs. However, reprogramming of the *Nanog* null MEFs either was 100-fold less efficient than WT MEFs (Carter et al., 2014) or required the inclusion of VitC in the reprogramming conditions (Schwarz et al., 2014). Taken together, these results suggested that the absence of *Nanog* presents a significant reprogramming roadblock.

**Figure 6:**
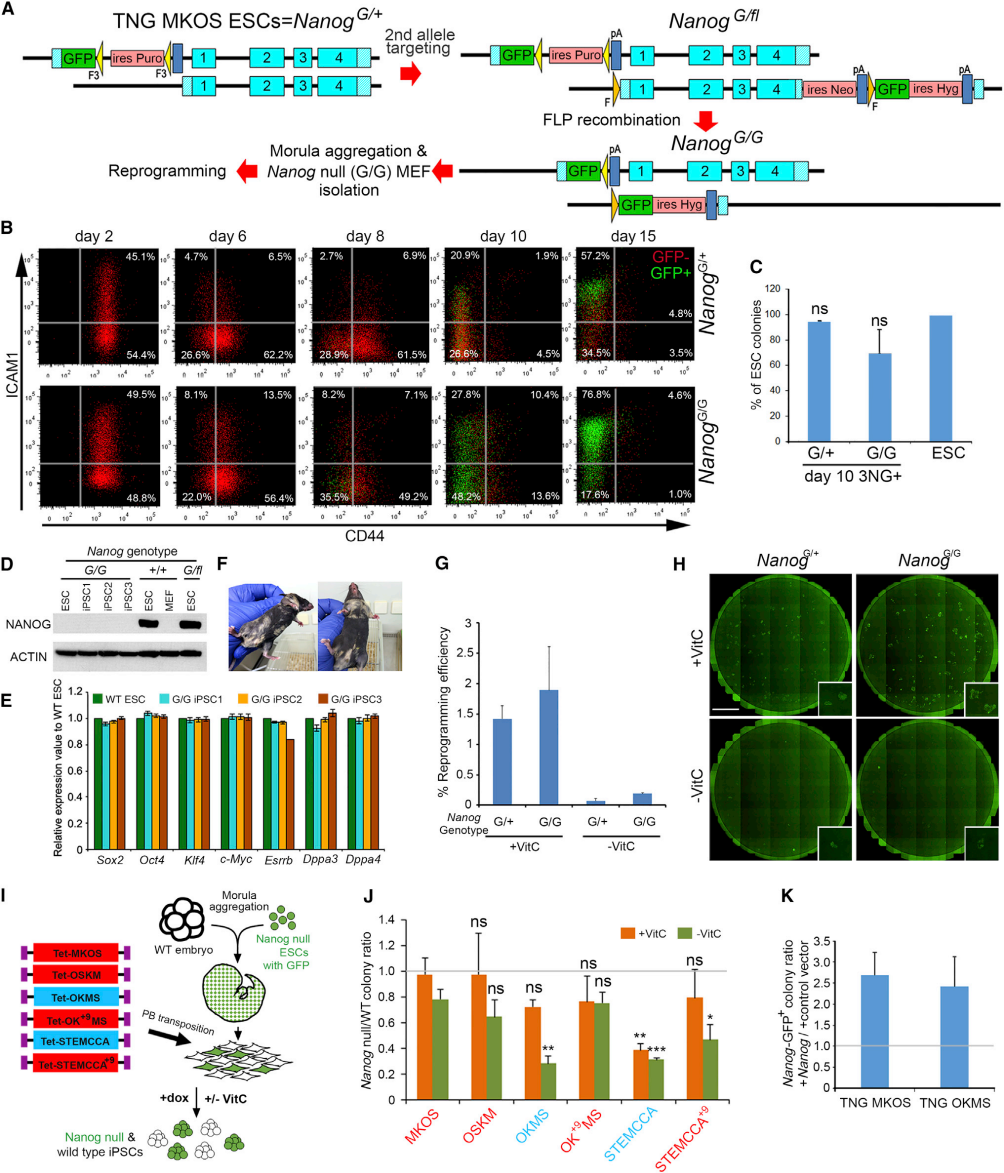
Reprogramming Cassette-Dependent Efficient *Nanog* Null MEF Reprogramming (A) A strategy for *Nanog* null TNG MKOS MEF reprogramming. The WT *Nanog* locus in TNG MKOS ES cells was converted to a Frt floxed allele via gene targeting, resulting in *Nanog*^*G/fl*^ ES cells. The remaining *Nanog* coding sequence was excised by transient expression of FLP, resulting in *Nanog* null MKOS ESCs (Nanog^G/G^). Blue boxes 1–4 indicate exons 1–4. *FLP* recombination target sites FRT (F) and F3 in the cell lines are indicated in orange and yellow, respectively. (B) CD44/ICAM1 expression changes during *Nanog*^*G/+*^ (TNG) and *Nanog*^*G/G*^ (null) MKOS MEF reprogramming. Red indicates *Nanog*-GFP^−^, and green indicates *Nanog*-GFP^+^. (C) *Nanog*-GFP^+^ CFA of 3NG^+^ cells sorted on day 10 of *Nanog*^*G/+*^ and *Nanog*^*G/G*^ MKOS MEF reprogramming. Cells were cultured in the presence of doxycycline for 10 days after the sorting. Error bars represent SD; n = 3 independent experiments. ns, not significant compared to ESCs, by Student’s t test. (D) The absence of Nanog protein in *Nanog*^*G/G*^ MKOS ESCs and *Nanog*^*G/G*^ iPSC lines was confirmed by western blotting. (E) qRT-PCR analysis of pluripotency genes in *Nanog*^*G/G*^ iPSC lines in comparison to a WT ESC line. (F) Chimeric mice generated with *Nanog*^*G/G*^ iPSC cell lines. (G) *Nanog*^*G/+*^ and *Nanog*^*G/G*^ MKOS MEFs were reprogrammed in the presence or absence of VitC (+VitC and -VitC, respectively). Reprogramming efficiency on day 15 was calculated as shown in Figure S2B. Error bars represent SD; n = 4–6 independent experiments. (H) Whole well images of *Nanog*^*G/+*^ and *Nanog*^*G/G*^ MKOS MEF reprogramming in the presence or absence of VitC (+VitC and -VitC, respectively). Scale bar, 7 mm. (I) A strategy for *Nanog* null MEF reprogramming with various reprogramming cassettes with *Klf4*_*L*_ (red) or *Klf4*_*S*_ (blue). WT and *Nanog* null mixed MEFs isolated from E12.5 chimeric embryos, generated with a *Nanog* null ESC line BT12 constitutively expressing GFP, were reprogrammed via PB transposons. dox, doxycycline. (J) Numbers of DPPA4^+^ colonies on day 15 were scored, and reprogramming efficiencies of *Nanog* null cells against WT cells were shown. Error bars represent SD; n = 3. ^∗∗^p < 0.01; ^∗∗∗^p < 0.001, ns, not significant compared to +VitC or -VitC MKOS reprogramming, respectively, by Student’s t test. (K) Increased reprogramming efficiency by *Nanog* overexpression during TNG MKOS or OKMS reprogramming. Error bars represent SD; n = 3 independent experiments. See also Figure S2B.

To elucidate reprogramming barriers associated to *Nanog* deficiency, we first generated *Nanog*^−/−^ ESCs by targeting the remaining intact *Nanog* allele of the TNG MKOS ESCs, from which Nanog null (*Nanog*^*G/G*^) MKOS MEFs could be derived (Figure 6A; Figure S4A). We then induced expression of MKOS and analyzed ICAM1 and CD44 dynamic changes during reprogramming. Surprisingly, we could only detect a slight delay, if any, in CD44 downregulation but with accelerated *Nanog*-GFP expression, indicating activation of the *Nanog* promoter (Figure 6B). The CFA and *Dppa3* and *Dppa4* expression of *Nanog*^*G/G*^ 3NG^+^ (CD44^−^ICAM1^+^*Nanog*-GFP^+^) cells on day 10 was similar to that of *Nanog*^G/+^ 3NG^+^ cells, indicating that not only the cell surface markers but also the phenotypic characteristics are comparable (Figures 6C and S4C). We confirmed the absence of *Nanog* expression in the *Nanog*^*G/G*^ iPSC lines, as well as pluripotency gene expression (Figures 6D and 6E). Chimera contribution demonstrated that the *Nanog*^*G/G*^ iPSCs were indeed pluripotent (Figure 6F). *Nanog*^*G/G*^ MKOS MEFs produced as many *Nanog*-GFP^+^ colonies as TNG MKOS MEFs, not only in the presence but also in the absence of VitC (Figures 6G, 6H, and S4B). Despite displaying slightly increased *Nanog*-GFP expression, which could be due to the two *Nanog*-GFP alleles in *Nanog*^*G/G*^ cells and/or lack of the auto-repression by NANOG protein (Fidalgo et al., 2012; Navarro et al., 2012), our results demonstrate that there is no clear additional roadblock in *Nanog* null MKOS MEF reprogramming. Thus, the previously described *Nanog* null reprogramming phenotypes might have been a product of the reprogramming systems used in these studies.

### Cassette-Dependent Phenotypes of *Nanog* Null MEF Reprogramming

To date, all the reported *Nanog*^−/−^ MEF reprogramming experiments have been carried out with the STEMCCA reprogramming cassette or four retroviral vector reprogramming systems (Carter et al., 2014; Schwarz et al., 2014; Silva et al., 2009), both of which tend to generate a high proportion of partially reprogrammed cells, unlike MKOS cassette-mediated reprogramming. To further investigate whether our ability to efficiently reprogram *Nanog*^−/−^ MEFs was due to the MKOS reprogramming cassette, we performed *Nanog* null MEF reprogramming using PB transposons with various polycistronic cassettes described earlier. A *Nanog* null ESC line constitutively expressing GFP, RCNβH-B(t), was used to obtain *Nanog* null MEFs through morula aggregation (Chambers et al., 2007). A mixture of GFP^+^*Nanog* null and GFP^−^ WT MEFs harvested from chimeric embryos was reprogrammed in the presence or absence of VitC (Figure 6I). To assess reprogramming efficiency, cells were stained for DPPA4 after 15 days of doxycycline administration, and relative reprogramming efficiencies of WT and *Nanog* null MEFs were calculated by normalizing the DPPA4^+^ colony numbers by the numbers of GFP^−^ WT and GFP^+^*Nanog* null MEFs on day 0 (Figure 6J). Even in this primary PB reprogramming setting, the lack of *Nanog* did not significantly affect the reprogramming efficiency in either the presence or the absence of VitC when the MKOS or OSKM cassette was used (Figure 6J). Similarly, *Nanog*^−/−^ MEFs reprogrammed with the OKMS cassette did not show an evident reduction of iPSC colony formation in comparison to WT MEFs in the presence of VitC (Figure 6J). However, the reduction of reprogramming efficiency became severe in the absence of VitC, ending up to 70% lower than WT (Figure 6J). Interestingly, the STEMCCA cassette could not overcome the lack of *Nanog* even in the presence of VitC, wherein *Nanog* null MEFs had 60%–70% reprogramming efficiency reduction (Figure 6J). Surprisingly, these reduced reprogramming efficiencies of *Nanog*^−/−^ cells with the OKMS and STEMCCA cassettes were almost completely or partially rescued with OK^+9^MS and STEMCCA^+9^, respectively (Figure 6J). In summary, *Nanog* expression appeared more critical in less efficient reprogramming conditions. Absence of endogenous *Nanog* had little impact on reprogramming, either in the presence or absence of VitC, when more efficient polycistronic reprogramming cassettes with *Klf4*_*L*_ were used. Comparing reprogramming efficiency between WT morula-derived MEFs and *Nanog* null ESC-derived MEFs that did not go through germline may not be a perfect setup. However, the previous works had taken similar approaches and concluded that endogenous *Nanog* was critical for efficient reprogramming, even though it was not absolutely essential for the iPSC generation (Carter et al., 2014; Schwarz et al., 2014). Thus, previous results reporting *Nanog* importance in the iPSC generation were probably due to inefficient and/or less homogenous reprogramming systems, such as viral delivery of the reprogramming factors or use of the polycistronic cassette with *Klf4*_*S*_. Notably, addition of exogenous *Nanog* expression could increase the reprogramming efficiency in both TNG MKOS and TNG OKMS reprogramming systems, in agreement with previous observations in various reprogramming systems (Figures 6K and S4D) (Hanna et al., 2009; Silva et al., 2009). Therefore, the molecular mechanisms of reprogramming enhancement both by endogenous *Nanog* in inefficient reprogramming systems and by exogenous *Nanog* even in efficient reprogramming systems are of interest.

## Discussion

To elucidate the precise molecular mechanisms of reprogramming and improve its efficiency and kinetics, it is critical to generate molecular route maps and identify roadblocks to regain pluripotency. Several different reprogramming systems have been used for this purpose. In this work, we have reported that polycistronic reprogramming cassettes with the four Yamanaka factors in different orders have distinct potentials to produce fully reprogrammed cells as well as cells trapped in partially reprogrammed states due to distinct severity and/or type of molecular obstacles.

### Reprogramming Routes

Using the MKOS and OKMS cassettes, we have demonstrated that the pattern of E-CAD, CD44, and ICAM1 expression changes and the gene expression profiles of the intermediates can vary largely depending on the reprogramming system. Does this mean that cells reach a pluripotent state through different routes? While PCA clearly distinguished MKOS from OKMS intermediates (2NG^−^ and 3NG^−^), flow cytometry analysis of the sorted populations demonstrated that E-CAD^+^ 3NG^−^ was the major source of *Nanog*-GFP^+^ cells in both MKOS OKMS reprogramming. In addition, E-CAD^+^ 2NG^−^ cells gave rise to E-CAD^+^ 3NG^−^ cells more efficiently than any other intermediates. Therefore, it is likely that, during both MKOS and OKMS reprogramming, the cells that finally become iPSCs display the same changes in E-CAD, CD44, and ICAM1 expression, i.e., transition from E-CAD^+^ 2NG^−^ to E-CAD^+^ 3NG^−^ before expressing *Nanog*-GFP. However, the majority of the reprogramming intermediates in the OKMS system was trapped in partially reprogrammed states and prevented the identification of cells progressing toward the iPSC state, concealing critical gene expression changes during iPSC generation. These data highlight the importance of using reprogramming systems with less heterogeneity and high efficiency to characterize the cells that become iPSCs.

### System-Dependent Roadblocks and Dispensability of *Nanog*

There are two kinds of gene expression changes during reprogramming: those that are necessary to establish the pluripotent state and those that do take place but do not affect the generation of iPSC. Changes of the first group, which are difficult to achieve and occur at a lower frequency and/or with slower kinetics are considered here as molecular roadblocks. Upregulation of E-CAD and some of the transcription factors shown in Figures S2C and S2D could belong to the first class in OKMS reprogramming systems. *Nanog* upregulation clearly belongs to the first group in OKMS and STEMCCA reprogramming; however, it falls into the second group when more efficient reprogramming cassettes with *Klf4*_*L*_ are used. It is likely that cells with less *Klf4* expression depend on *Nanog* to overcome some obstacles and establish the pluripotency gene expression network, while abundant *Klf4* is sufficient to compensate for a lack of *Nanog*. It is also notable that *Nanog*^−/−^ epiblast stem cells (EpiSCs) can be reprogrammed more efficiently by *Esrrb* overexpression rather than *Klf4* expression (Festuccia et al., 2012), suggesting several alternative routes to iPSCs. While the indispensability of *Nanog* for iPSC generation is system and context dependent, overexpression of *Nanog* improved reprogramming efficiency even in the efficient MKOS reprogramming system. In addition, the importance of *Nanog* could potentially be significant in less efficient reprogramming systems such as human cell reprogramming. Thus, the roles of *Nanog* in reprogramming are still of interest.

### Reprogramming Factor Stoichiometry

The generation of iPSCs is accomplished by forced gene expression changes resulting from overexpression of *Oct4, Sox2, Klf4* and *c-Myc*: O, S, K, and M, respectively. Therefore, it is reasonable that reprogramming efficiencies and gene expression changes largely depend on expression levels of each factor and/or the balance of four factors, i.e., stoichiometry. Replacement of *Klf4*_*S*_ with *Klf4*_*L*_ results in higher KLF4 protein level and improved reprogramming efficiency. This made the reprogramming process traceable by monitoring CD44 and ICAM1 expression and diminished Nanog dependency. Consistent with these findings, it has been recently reported that transcription pause release is a rate-limiting step for iPSC generation and that KLF4 facilitates the recruitment of P-TEFb, a positive transcription elongation factor, at the pluripotency gene loci (Liu et al., 2014). Moreover, the combined transfection of *Klf4* and activated *Stat3* was sufficient to bypass the necessity for *Nanog* during EpiSCs reprogramming (Stuart et al., 2014). Our rescue strategy did not distinguish whether the stoichiometry of the four factors, the absolute KLF4 levels, or both were critical for efficient reprogramming. Therefore, future work with different experimental settings is necessary to address this point.

### Optimal Tools for Mechanistic Analyses

Some polycistronic cassettes, such as MKOS and OSKM, produce robust reprogramming progression and minimize generation of partially reprogrammed cells. Therefore, they are more suited for investigating molecular events important for progression toward a pluripotent state. On the other hand, analysis of partially reprogrammed cell populations from less efficient reprogramming systems should provide information on context-dependent roadblocks and their effects. The impact of distinct reprogramming cassettes on the reprogramming phenotypes might also be different depending on cell types, species, culture conditions, gene delivery methods, or possibly even different expression levels of endogenous *Klf4*. Overall, our work highlights the importance of taking into account the reprogramming systems used when investigating the molecular mechanisms of cellular reprogramming.

## Experimental Procedures

All animal experiments were approved by the University of Edinburgh Animal Welfare and Ethical Review Body, performed at the University of Edinburgh, and carried out according to regulations specified by the Home Office and Project License 60/4435.

### Cell Culture

MEFs with 129 genetic backgrounds were prepared from embryonic-day (E)12.5 embryos as described before (Kaji et al., 2009) and cultured in MEF medium (Glasgow minimun essential medium [GMEM] supplemented with 10% fetal calf serum [FCS], penicillin-streptomycin, 1× non-essential amino acids) (Invitrogen), 1 mM sodium pyruvate, 2 mM glutamine, 0.05 mM 2-mercaptoethanol (Life Technologies) supplemented with 5 ng/ml fibroblast growth factor-2 (FGF2) and 1 ng/ml heparin. ESCs and iPSCs were cultured in ESC medium (MEF medium supplemented with human LIF [leukemia inhibitory factor], 100 U/ml).

### *piggyBac* Transposon Reprogramming

*piggyBac PB-TAP IRI attP2LMKOSimO*, *PB-TAP IRI 2LOSKMimO*, and *PB-TAP IRI tetO-STEMCCAimO* have been described previously (dos Santos et al., 2014; O’Malley et al., 2013). The *piggyBac* OKMS reprogramming vector, *PB-TAP IRI 2L OKMSimO*, was generated by replacing the OSKM cassette of *PB-TAP IRI 2LOSKMimO* with the OKMS cassette (Kim et al., 2015). Replacement of *Klf4*_*s*_ with *Klf4*_*L*_ was conducted by using the Gibson assembly. *Rosa*^*rtTA/rtTA*^, *Nanog*^*eGFP/+*^ MEFs, or MEFs derived from chimeric embryos generated with *Nanog*^−/−^ BT12 ESCs were plated in a six-well plate at 1 × 10^5^ MEFs per well. The following day, the PB transposons carrying a reprogramming cassette (500 ng) and the CAG-promoter-driven *rtTA* (500 ng), plus the CMV-promoter-driven-*HyPBase* (500 ng) in the case of BT12 ESC-derived *Nanog*^−/−^ MEFs, were introduced into the MEFs using Fugene HD (Promega) (Yusa et al., 2011). The cells were cultured in reprogramming medium for 15–16 days. Plasmid sequences are available upon request.

### Tg MEF Reprogramming

TNG MKOS/OKMS and *Nanog* null MKOS MEFs were isolated from E12.5 chimeric embryos generated via morula aggregation. One-tenth of the dissociated cells were exposed to doxycycline (1,000 ng/ml) for 2 days, and mOrange expression was measured by flow cytometry to assess the proportion of Tg MEFs. For reprogramming experiments, Tg MEFs were diluted to 5% by the addition of WT MEFs and plated in a gelatinized six-well plate at 1 × 10^5^ cells per well (5,000 Tg MEFs per well). For sorting experiments, MEFs were plated at 2 × 10^5^ cells per gelatinized 100-mm plate with 5% Tg MEFs. All reprogramming experiments were carried out in the aforementioned ESC medium supplemented with 1.0 μg ml^−1^ doxycycline (Sigma) and 10 μg/ml VitC (Sigma) unless otherwise specified. Medium was changed every 2 days. Whole-well imaging and quantification of total and GFP^+^ colony numbers were performed with the Celigo S Cell Cytometer (Nexcelom). Overexpression of *Nanog* during TNG MKOS/OKMS MEF reprogramming was performed with *FUW-TetO-Nanog* (Addgene #40800) (Buganim et al., 2012).

### Flow Cytometry and Cell Sorting

The following antibodies from eBioscience were used with the indicated dilution: ICAM1-biotin (13-0541; 1/100), CD44-eFluor 450 (48-0441-82; 1/100), CD44-allophycocyanin (APC) (17-0441; 1/300), streptavidin-phycoerythrin (PE)-Cy7 (25-4317-82; 1/1500), and E-CADHERIN-eFluor 660 (50-3249-80; 1/200). Dead cells were excluded using DAPI (Invitrogen, 0.5 ng ml^−1^) or DRAQ5 (eBioscience, 10 μM) nucleic acid staining. Cells were treated with 0.25% trypsin and 1 mM EDTA (Life Technologies) for 1–2 min at 37°C, collected in GMEM containing 10% FCS, and counted. Staining was carried out in FACS (fluorescence-activated cell sorting) buffer (2% FCS in PBS) for 30 min at 4°C, and followed by washing with FACS buffer, sorting, and/or analysis with FACSAriaII or LSRFortessa (BD Biosciences). Excitation laser lines and filters used for each fluorophore are summarized in Table S1. Data were analyzed using FlowJo software (Tree Star). For colony formation assays, sorted cells were plated on γ-irradiated MEFs in 12-well plates at 1.0–3.0 × 10^3^ cells per well. *Nanog*-GFP^+^ colonies were quantified 10 days after sorting. For time course analysis of the sorted subpopulation, the cells were plated on γ-irradiated MEFs in 48-well plates at 3 × 10^4^ cells per well. In both cases, cells were cultured in reprogramming medium after sorting.

### Immunofluorescence

Cells were fixed with 4% paraformaldehyde (PFA) (Sigma) for 10 min; permeabilized with 0.1% Triton X/PBS for 1 hr at room temperature; blocked with 5% Normal Goat Serum (SouthernBiotech), 0.1% Tween 20 in PBS for 1 hr; and stained overnight with the primary antibody at 4°C (1:1,000 in blocking solution) and 1 hr with the secondary antibodies (1:1,000 in blocking solution). Anti-mouse NANOG and DPPA4 antibodies were obtained from eBioscience (14-5761) and Cosmo Bio (CAC-TMD-PB-DP4), respectively.

### Western Blotting

Human embryonic kidney (HEK) cells (2 × 10^6^ cells per 100-mm plate) were seeded in MEF medium 1 day before transfection. The following day, cells were transfected using Lipofectamine 2000 (#11668027, Life Technologies) with the PB transposons carrying the *rtTA* and the appropriate cassette (12 μg each). One day after transfection, the medium was replaced with MEF medium containing 1,000 ng/ml doxycycline. At 24 hr later, the cells were harvested and re-suspended in hypotonic buffer (10 mM HEPES, pH 7.9, 1.5 mM MgCl_2_, 10 mM KCl, 0.2 mM PMSF, 0.5 mM DTT). The swollen cells were lysed with 1% NP-40, and nuclei were pelleted and then re-suspended in hypertonic buffer (20 mM HEPES, pH 7.9, 25% glycerol, 1.5 mM MgCl_2_, 1 M NaCl, 0.2 mM PMSF, 0.5 mM DTT). The extracted soluble nuclear proteins were collected after centrifugation. Protein concentration was assessed with the Bio-Rad protein assay, and 10 μg of each sample was used for SDS-PAGE. For the western blotting, rabbit anti-KLF4 (1:500; sc-20691, Santa Cruz Biotechnology), rabbit anti-LAMIN B1 (1:1,000; ab16048, Abcam), anti-rabbit immunoglobulin G-horseradish peroxidase (IgG-HRP) secondary antibodies (1:5,000; Sigma-Aldrich), and the ECL Western Blotting Detection Kit (GE Healthcare) were used.

### Global Gene Expression Analysis

Multiplexed RNA sequencing was performed as described previously using 10 ng RNA of each sorted subpopulation, and quality control and alignment to the mouse reference genome (NCBI37.1/mm9) were performed using the STRT method pipeline as previously described (Islam et al., 2011). The heatmaps were generated using the average of replicates of variance-stabilizing transformed (VST) gene expression read counts (generated using the DESeq package in R), and mean-centered per gene. Color code black (0.00) means that the expression level is equal to the mean expression levels of all samples (from MEFs to WT ESCs) of the gene. PCA was performed in R and plotted with the scatterplot3d library (Ligges and Maechler, 2003). Cluster overlaps were plotted using a Circos-generated chord diagram (Krzywinski, et al., 2009).

## Author Contributions

E.C., S.M., and K.K. generated the TNG MKOS/OKMS ESC lines, and S.M. generated the *Nanog* null MKOS ESC line. E.C. characterized MKOS/OKMS reprogramming. E.C. and S.M. characterized *Nanog* null MEF reprogramming. S.S. analyzed RNA-sequencing data. A.J. and S.L. performed multiplexed RNA sequencing and collected data. S.K. and K.W. generated the OKMS cassette and identified the difference of *Klf4* in the publicly available polycistronic reprogramming cassettes. I.C. generated the TNG ESC line and contributed to the manuscript. E.C. and K.K. conceived the study and wrote the manuscript.

## Accession Numbers

The accession number for the gene expression datasets reported in this paper is GEO: GSE70816.

## Supplemental Information

Document S1. Supplemental Experimental Procedures, Figures S1–S4, and Tables S2 and S3

Table S1. Classification of DEGs in MKOS or OKMS Reprogramming Based on the Expression Dynamics, Related to Figure 4

Document S2. Article plus Supplemental Information
