# Supplementary material for: Reprogramming Roadblocks Are System Dependent
Source: Stem Cell Reports. 2015 Aug 13;5(3):350–64. doi: 10.1016/j.stemcr.2015.07.007 (PMC4618455; doi:10.1016/j.stemcr.2015.07.007)
Supplement: Document S1. Supplemental Experimental Procedures, Figures S1–S4, and Tables S2 and S3 [file mmc1.pdf]

Stem Cell Reports, Volume *5*

Supplemental Information

Reprogramming Roadblocks Are System Dependent

Eleni Chantzoura, Stavroula Skylaki, Sergio Menendez, Shin-Il Kim, Anna Johnsson,

Sten Linnarsson, Knut Woltjen, Ian Chambers, and Keisuke Kaji

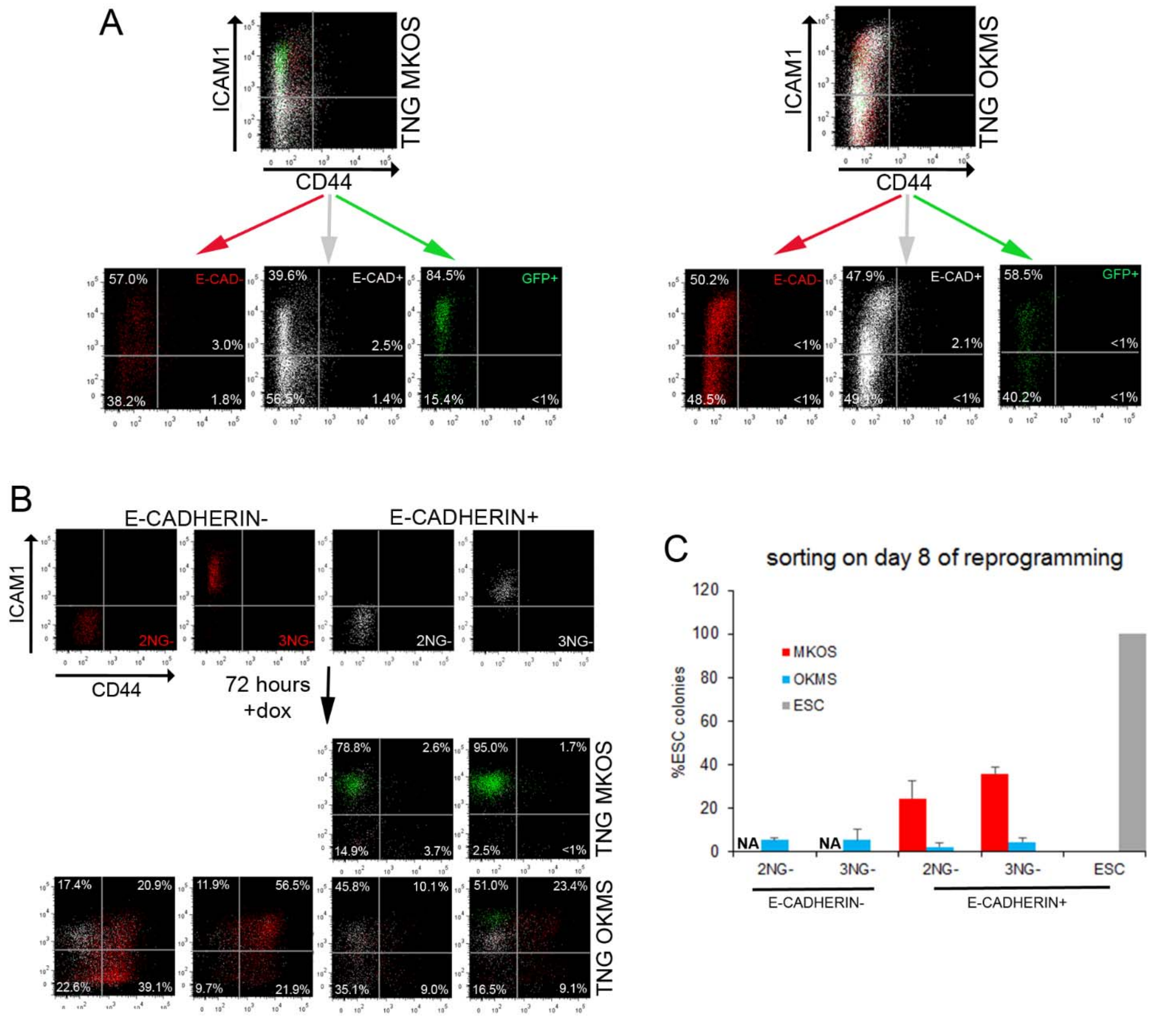

Figure S1, related to Figure 3. TNG MKOS/OKMS cells sorted on day10 of reprogramming. A. Pre-sorting MKOS/OKMS reprogramming intermediates on day 10. **B.** E-CAD, CD44, ICAM1 and Nanog-GFP expression 72 hours after sorting of MKOS E-CAD^{+} 2NG^{-} and 3NG^{-} cells, OKMS E-CAD$^{-/+}$ 2NG^{-} and 3NG^{-} cells in reprogramming conditions. **C.** E-CAD-/+ 2NG-, 3NG- cells as well as ESCs were seeded at clonal density on day 8 of reprogramming, and Nanog-GFP+ iPSC colonies were counted after 10 days of further culture. The graph depicts the relative probability of each subpopulation to generate colonies compared to sorted ESCs. Error bars represent s.d. n = 3 independent experiments.

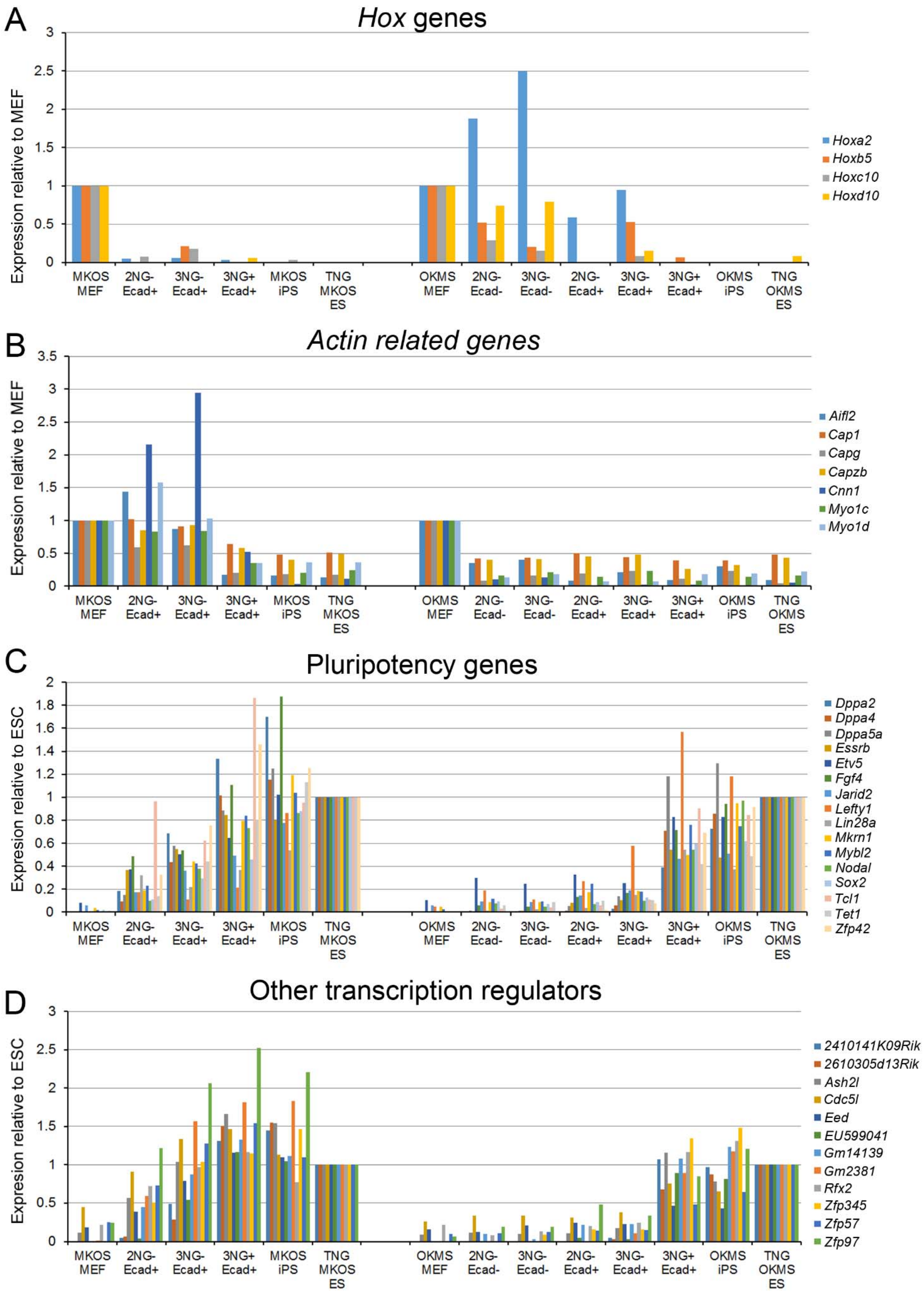
A
Hox genes
Expression relative to MEF
Hoxa2
Hoxb5
Hoxc10
Hoxd10
MKOS MEF
2NG-Ecad+
3NG-Ecad+
3NG+Ecad+
MKOS iPS
TNG MKOS ES
OKMS MEF
2NG-Ecad-
3NG-Ecad-
2NG-Ecad+
3NG-Ecad+
3NG+Ecad+
OKMS iPS
TNG OKMS ES
B
Actin related genes
Expression relative to MEF
Aifl2
Cap1
Capg
Capzb
Cnn1
Myo1c
Myo1d
C
Pluripotency genes
Expresion relative to ESC
Dppa2
Dppa4
Dppa5a
Essrb
Etv5
Fgf4
Jarid2
Lefty1
Lin28a
Mkrn1
Mybl2
Nodal
Sox2
Tcl1
Tet1
Zfp42
D
Other transcription regulators
Expresion relative to ESC
2410141K09Rik
2610305d13Rik
Ash2l
Cdc5l
Eed
EU599041
Gm14139
Gm2381
Rfx2
Zfp345
Zfp57
Zfp97

Figure S2, related to Figure 4. Expression of DEGs cross-classified in MKOS and OKMS reprogramming. **A.** *Hox* genes in the MA_OB DEGs. **B.** *Actin* related genes in the MB_OA DEGs **C.** Pluripotency genes in the MD_OE DEGs. **D.** Other transcription regulators in the MD_OE DEGs. Data represent an average of replicates in the RNA-sequencing, normalized with the expression values of MEFs or ESCs. Raw expression values are available in Supplemental Table S1.

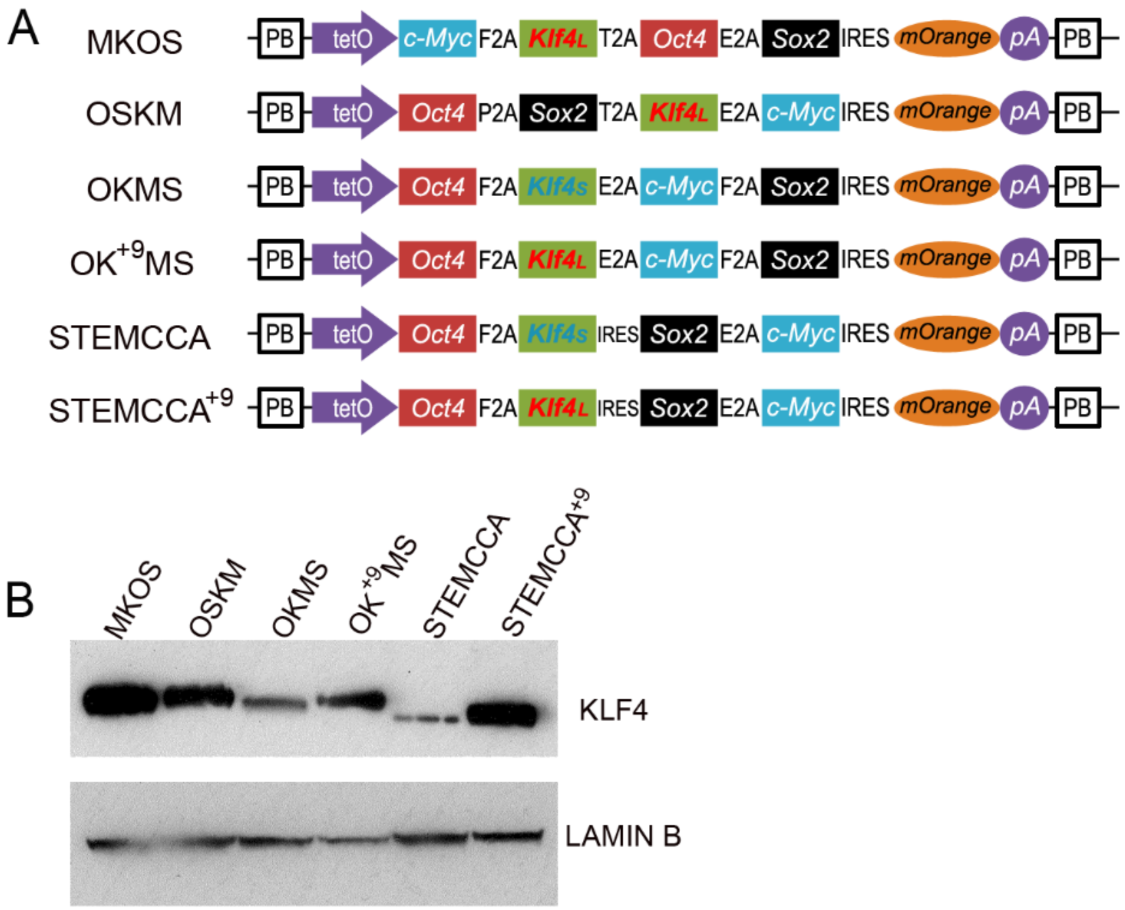

Figure S3, related to Figure 5. Polycistronic reprogramming cassettes with Klf4$_L$ and Klf4$_S$. A. The MKOS and OSKM cassettes carry Klf4$_L$. Klf4$_S$ in the OKMS and STEMCCA cassettes were replaced with Klf4$_L$, resulting the OK^{+9}MS and STEMCCA^{+9} cassettes. **B.** The polycistronic reprogramming cassettes with Klf4$_L$ yield more KLF protein than those with Klf4$_S$.

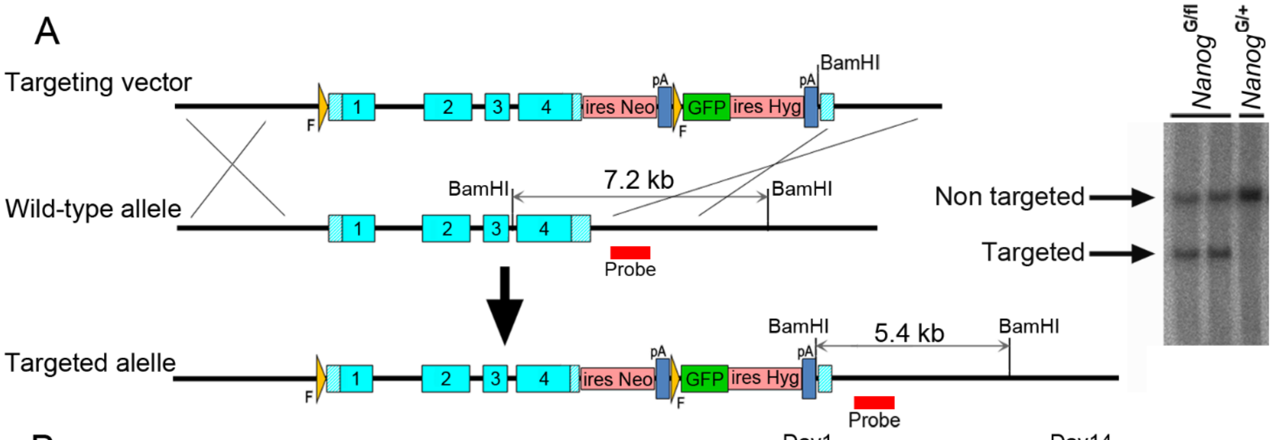

B

	Day1		Day14					
+VitC	mOrange+ cell No.		GFP+ colony No.		Rep efficiency %		Avarage	STDEV
G/+ #1	6,995	6,703	83	88	1.187	1.313	1.419	0.216
G/+ #2	6,778	6,443	111	107	1.638	1.661		
G/+ #3	8,620		112		1.299			
G/G #2	4,459	3,554	104	95	2.332	2.673	1.894	0.717
G/G #5	6,163	6,536	81	82	1.314	1.255		

	Day1		Day14					
-VitC	mOrange+ cell No.		GFP+ colony No.		Rep efficiency %		Avarage	STDEV
G/+ #1	22,207	20,174	12	14	0.054	0.069	0.072	0.043
G/+ #2	19,886	18,927	22	24	0.111	0.127		
G/+ #3	26,841	25,817	9	9	0.034	0.035		
G/G #4	18,251	15,284	22	42	0.121	0.275	0.190	0.011
G/G #6	13,918	12,918	26	23	0.187	0.178		

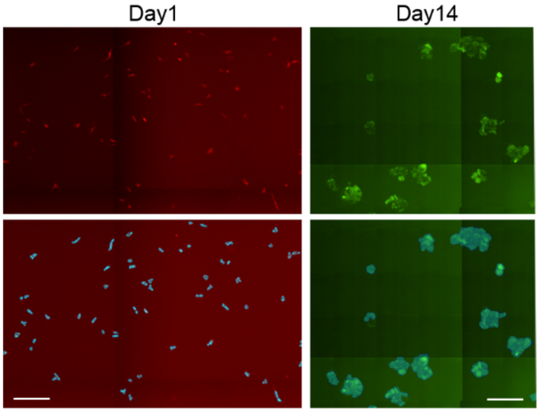

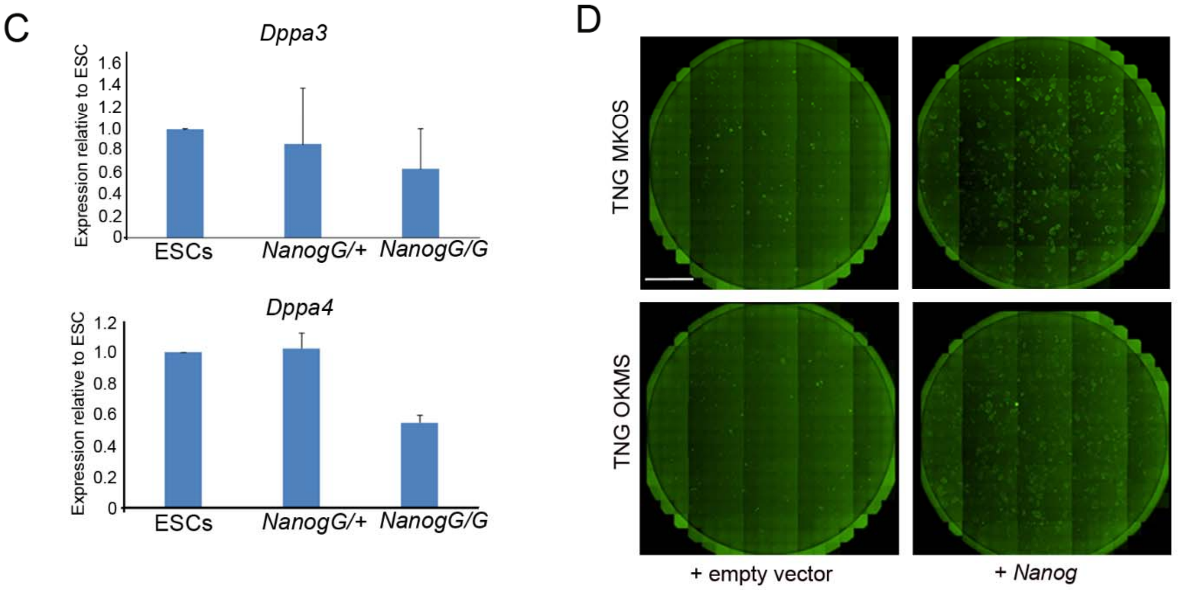

Figure S4, related to Figure 6. Reprogramming in the absence of Nanog, or with overexpression of Nanog. A. The targeting strategy of the remaining wild-type *Nanog* allele in the TNG MKOS ESC line, preceding FLP excision. BamHI genome digestion yielded untargeted 7.2 kb and targeted 5.4 kb fragments detected by the probe indicated as a red bar. Blue boxes = exon 1-4. F = Frt **B.** Raw data from $Nanog^{G/+}$ and

Nanog$^{G/G}$ MKOS MEF reprogramming experiments in Figure 6B. Each batch of MEFs was used in duplicates except G/G #3. To make the reprogramming efficiency calculation as accurate as possible, numbers of mOrange^{+} cells were counted at day1 of reprogramming via Celigo as shown with blue segmentation lines. Scale bar, 50 μm (Day 1), 1 mm (Day 14). Nanog-GFP^{+} colony numbers counted by Celigo at day14 (filled with blue) were divided with mOrange+ cell numbers to calculate reprogramming efficiency (Rep efficiency %). **C.** Dppa3 and Dppa4 expression in *Nanog*$^{G/+}$ and *Nanog*$^{G/G}$ 3NG+ cells sorted on day 10 of reprogramming. Relative expression levels to ESC are shown. Error bars represent s.d., n = 3 independent experiments. **D.** Nanog over expression in TNG MKOS/OKMS MEF reprogramming. Lentiviral empty or Nanog expression vector was infected at day 1 of reprogramming. Whole well images on day 15 are shown. Scale bar, 7 mm.

Table S1, related to Figure 4. Classification of DEGs in MKOS or OKMS reprogramming based on the expression dynamics.

Table S2. Excitation laser lines and filters for each fluorophore

LSR Fortessa		Excitation Laser			
		405 nm	488 nm	561 nm	640 nm
filter	450±40	eFluor450			
	530±30		GFP		
	582±15			mOrange	
	780±60			PECy7	
	670±30				eFluor660

FACS ARIA II		Excitation Laser			
		405 nm	488 nm	561 nm	640 nm
filter	450±40	eFluor450			
	525±50		GFP		
	582±15			mOrange	
	780±60			PECy7	
	670±14				eFluor660

Table S3, related to Figure 6. RT-PCR primers

Gene name	Primer sequence
Sox2	F- TCTGTGGTCAAGTCCGAGGC<br>R- TTCTCCAGTTCGCAGTCCAG
Oct4	F- CCAACGAGAAGAGTATGAGGC<br>R- GTGCTTTTAATCCCTCCTCAG
Klf4	F- GGCGAGAAACCTTACCACTGT<br>R- TACTGAACTCTCTCTCCTGGCA
c-Myc	F- TCAAGCAGACGAGCACAAGC<br>R- TACAGTCCCAAAGCCCCAGC
Esrrb	F- TGGCAGGCAAGGATGACAGA<br>R- TTTACATGAGGGCCGTGGGA
Dppa3	F- AGATAGGATGCACAACGATCC<br>R- AAAATGCTTTTATTACAAATTTCTGGA
Dppa4	F- TGCAAAGGCTAAAGCAACG<br>R- TTTTCCCTCCTTTGGTTTACAA
Tbp	F- GGGGAGCTGTGATGTGAAGT<br>R- CCAGGAAATAATTCTGGCTCA

Supplemental Experimental Procedures

***Sp3* targeting vector construction and the generation of the TNG MKOS/OKMS ES lines.** The integration site of PB-TAP IRI attP2LMKOSimO piggyBac (PB) transposon in the D6s4B5 iPSC line, the *Sp3* locus, has been identified by splinkerette PCR (Horn et al., 2007). To generate a targeting vector which enables the integration of a doxycycline (Dox)-inducible reprogramming cassette in the *Sp3* locus of any ES cell line, a 14.6kb DNA fragment from the *Sp3* locus was retrieved, and an *attR1-CmR-attR2* cassette was inserted in the 3rd intron of the *Sp3* gene for Gateway cloning (*Sp3 DTA Amp attR1R2*). A CAG promoter-driven *rtTA* expression cassette has been inserted adjacent to the Gateway cloning site resulting in *Sp3 DTA Amp attR1R2 rtTA*. A ~15kb fragment containing partial PB 3' TR, chicken b globin insulator, Dox-inducible MKOS/OKMSimO reprogramming cassette, Lamin B2 replicator, chicken β-globin insulator, partial PB 5' TR , as well as a PGK promoter-driven neomycin resistance cassette and a MC1 promoter-driven *HSV-TK* cassette floxed by FRT sites (NeoTk cassette), was inserted into the *Sp3 DTA Amp attR1R2* through Gateway cloning. The vector maps and sequences are available upon request. The resulting targeting vector, *PB 2L MKOS/OKMSimO neotk rtTA Sp3*, was linearized with SgfI and then electroporated into TNG ESCs (Chambers et al., 2007). After G418 selection (400 μg/ml), correctly targeted clones were identified by Southern blotting and the NeoTk cassette was removed by transient FLP expression, resulting in TNG MKOS/OKMS ESC lines.

Generation of the Nanog null (NN) MKOS ESC line. The targeting vector for the Nanog second allele has been generated by replacing the *IRES-pac-C2-MAZ-loxP-eGFP-β-globin pA* cassette with the *IRES-Blasticidin S deaminase gene (Bsd)-BGH pA-FRT-eGFP-IRES-hygromycin resistance gene (Hyg)-BGH pA* cassette of the *Nanog*

conditional targeting vector (Chambers et al., 2007), after exchanging the loxP site before the exon 1 with a FRT. The resulting *pConditonal frt IB pA frt EGFP IH pA* vector was linearized with SpeI and electroporated into TNG MKOS ESCs. After identifying correctly targeted clones by Southern blotting, the remaining functional *Nanog* allele was removed via FLP expression. Loss of the *Nanog* gene and protein in the survived cells under hygromycin selection was confirmed by genomic PCR and Western blotting, and the bulk population was used as a *Nanog* null (NN) MKOS ES line.

Viral infection. For lentivirus preparation, $2x10^6$ HEK cells per 100 mm dish were seeded in MEF medium one day before transfection. 6 µg of lentiviral vector *FUW-TetO-Nanog* (Addgene #40800) or *FUW-TetO* generated from *FUW-TetO-Nanog* by removing *Nanog* were transfected into HEK293 cells together with a second generation packaging system *psPax2* (4.5 µg, Addgene #12260) and *pMD2.G* (1.5 µg, Addgene #12259) using the calcium phosphate method. DNA (12 µg in total in 427 µl water) was mixed first with 63 µl of 2 M $CaCl_2$, and then with 500 µl of 2x HBS buffer (0.28 M NaCl, 0.05 M HEPES, 1.5 mM Na_2HPO_4, pH7.0), before administration to the cells. Twenty-four hours after transfection, the medium was replaced with fresh MEF medium. After 24 hours, virus-containing supernatants were filtered through a 0.45 µm Whatman cellulose acetate filter and supplemented with 8 µg/ml polybrene (Nacalai Tesque). MEFs, containing 5% transgenic MEFs and seeded at $1x10^5$ cells per well in 6 well-plates one day before infection, were incubated in the virus/polybrene-containing supernatants overnight TNG MKOS/OKMS MEF reprogramming.

RT-PCR. RNA extraction was performed using the TRIzol reagent (Life Technologies, 15596018), and was followed by reversion transcription with the M-MLV reverse transcriptase from Promega (M1701). Brilliant II SYBR® Green QPCR Master Mix (Agilent, 600828) was used for the real time PCR reaction. Primer sequences are listed in Table S3.

Supplemental Reference

Horn, C., Hansen, J., Schnütgen, F., Seisenberger, C., Floss, T., Irgang, M., De-Zolt, S., Wurst, W., von Melchner, H., and Noppinger, P.R. (2007). Splinkerette PCR for more efficient characterization of gene trap events. Nat. Genet. 39, 933–934.
